# Atrial fibrillation and obstructive sleep apnea in African populations: uncovering a neglected association

**DOI:** 10.11604/pamj.2021.39.55.29000

**Published:** 2021-05-20

**Authors:** Jean Jacques Noubiap

**Affiliations:** 1 Centre for Heart Rhythm Disorders, University of Adelaide and Royal Adelaide Hospital, Adelaide, Australia

**Keywords:** Atrial fibrillation, obstructive sleep apnea, Africa

## Abstract

Atrial fibrillation (AF), the most common sustained arrhythmia, is one of the risk factors with the largest relative increase in attributed cardiovascular mortality in Africa. There are important knowledge gaps in the epidemiology of AF in Africa, along with inadequate service provision for cardiac arrhythmias including AF. This paper comments on the available data on the prevalence and correlates of obstructive sleep apnea (OSA) in patients with AF in Africa. Two studies from Tunisia revealed a high prevalence of OSA based on polysomnography (77% and 90%) among patients with AF. Patients with OSA were more likely to report snoring, were older and had longer AF duration compared to those without OSA. The implications of these findings are discussed. Furthermore, key points on the mechanisms underlying the association between AF and OSA, the impact of OSA on AF-related outcomes and the screening and management of OSA in patients with AF are highlighted.

## Commentary

Atrial fibrillation (AF), the most common sustained arrhythmia, is a growing global epidemic. There were 37.6 million individuals who were affected (0.51% of global population) in 2017, including 3.046 million new cases [1]. This represents a 33% increase in the prevalence of AF during the last two decades [1]. Atrial fibrillation is associated with substantial morbidity and mortality. Individuals with AF have a five-fold increased risk of stroke, three-fold increased risk of heart failure and almost two-fold increased risk of overall mortality [2,3]. It is estimated that 0.287 million deaths and 5976 million disability-adjusted life years were attributable to AF in 2017 [1]. Although the very few population-based studies conducted in Africa have suggested a low prevalence of AF in the general population [4], according to some projections there will be more people with AF in Africa than in either China, the United States, or India by 2050 [5]. Atrial fibrillation is one of the risk factors with the largest relative increase in attributed cardiovascular mortality in Africa [6]. Moreover, the economic cost of AF is likely to be substantial in African countries [7]. Several studies along with the recent report of the Pan African Society of Cardiology (PASCAR) on cardiac arrhythmia services in Africa (2011-2018) have shown that the management of AF in Africa is challenging, due to limited infrastructural, financial and human resources [4,7]. The important knowledge gaps in the epidemiology of AF on the continent also contributes to inadequate service provision.

**Figure 1:**
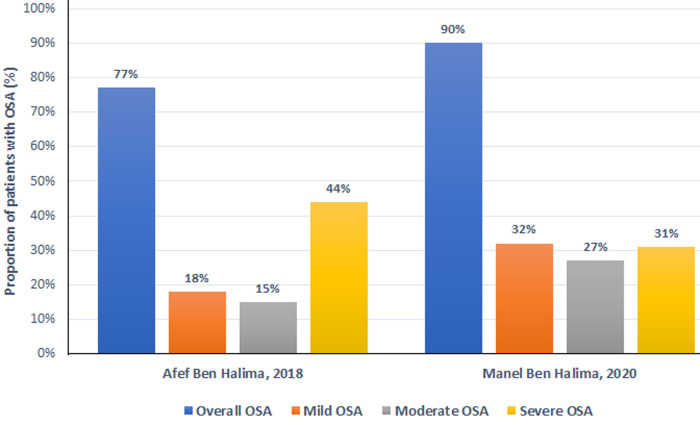
proportion of patients with obstructive sleep apnea; OSA: obstructive sleep apnea; overall OSA: Apnea-Hypopnea Index (AHI, in episodes per hour) ≥ 5; mild OSA: 5-14; moderate OSA: AHI 15-29; Severe OSA: AHI ≥ 30

Obstructive sleep apnea (OSA) is increasingly recognized as a major problem in patients with AF [8]. We performed a systematic search of PubMed and African Journals Online to identify studies on the prevalence and correlates of OSA in patients with non-valvular AF in Africa. Two studies were identified, both from Tunisia [9,10]. In the most recent one, Manel Ben Halima and colleagues included 100 patients with non-valvular AF, with a mean age of 66.4 years, 45% male (Table 1) [9]. According to the Berlin questionnaire, 64% of patients had a high probability of OSA (score ≥ 2). Based on polysomnography, 90% of patients had OSA (Apnea Hypopnea Index (AHI) ≥ 5 episodes/hour), including 32% with mild (AHI 5-14), 27% with moderate (AHI 15-29), and 31% with severe OSA (AHI ≥30). Factors independently associated with a diagnosis of OSA included age ˃ 61 years (adjusted odds ratio [aOR] 12.8, 95% confidence interval [CI]: 1.30-127.74; p = 0.029), AF duration > 2 years (aOR 6.4, 95% CI: 1.05-39.87; p = 0.044) and snoring (aOR 18.9, 95% CI: 1.62-221.13, p = 0.019) [9]. In the other study, Afef Ben Halima and colleagues reported on a group of 73 patients with AF and hypertension, with a mean age of 66.6 years and 21.9% of males [10]. The Berlin questionnaire identified 84% of patients with a high probability of OSA, whereas 77% (n = 56) of patients had a confirmed diagnosis of OSA (AHI ≥5) with polysomnography, including 18% with mild (AHI 5-14), 15% with moderate (AHI 15-29), and 44% with severe OSA (AHI ≥30) (Figure 1).

**Table 1:** characteristics of studies on obstructive sleep apnea in patients with atrial fibrillation in Africa

Characteristics	Afef Ben Halima, 2018				Manel Ben Halima, 2020			
	Total sample (n = 73)	Patients with OSA (n = 56)	Patients without OSA (n = 17)	P value	Total sample (n = 100)	Patients with OSA (n = 90)	Patients without OSA (n = 10)	P value
**Demographics**								
Age (years)	66.6 (10.7)	66.7 (10.9)	65.9 (10.2)	0.70	66.4 (9.7)	67.6 (8.6)	56.1 (13.0)	0.01
Males	21.9%	26.8%	5.9%	0.06	45%	45%	40%	0.70
**Anthropometry**								
BMI (kg/m2)	34.6 (6.5)	35 (6.8)	33.4 (5.0)	0.30	28.5 (4.8)	28.6 (4.9)	27.7 (3.9)	0.58
Neck circumference (cm)	38.8 (4.0)	NR	NR	NR	38.8 (4.9)	38.8 (4.9)	38.4 (4.8)	0.62
**Risk factors**								
Hypertension	100%	100%	100%	1.00	72%	73.3%	60%	0.37
Diabetes	26%	29%	18%	0.30	31%	28.8%	50%	0.17
Dyslipidemia	26%	26%	29%	0.70	45%	45.5%	40%	0.70
Tobacco smoking	19.2%	23%	6%	0.10	25%	26.6%	10%	0.47
**AF characteristics**								
AF duration (years)	4.5 (5.6)	4.7 (5.7)	4.1 (5.6)	0.50	4.4	4.4	4.1	0.02
Chronic AF	NR	NR	NR	NR	76%	77.8%	60%	0.21
Asymptomatic AF (EHRA I)	NR	NR	NR	NR	18%	15.6%	40%	0.06
**Symptoms**								
Snoring	NR	NR	NR	NR	87%	90%	60%	0.02
Sleep disorders	NR	NR	NR	NR	60%	62.2%	40%	0.17
Nocturia	NR	NR	NR	NR	56%	55.5%	60%	0.78
Daytime sleepiness	67.1%	68%	65%	0.90	80%	81%	70%	0.40
Epworth score	NR	7 (6)	5.4 (4.6)	0.30	10.3 (4.1)	10.5 (4.1)	9 (4.5)	0.20
Memory impairment	NR	NR	NR	NR	73%	74.4%	60%	0.33
Morning fatigue	NR	NR	NR	NR	66%	66.6%	60%	0.67
Cognitive impairment	16.4%	21%	0%	0.03	41%	42.2%	30%	0.45

AF: atrial fibrillation; BMI: body mass index; EHRA: European Heart Rhythm Association; OSA: obstructive sleep apnea; NR: not reported

Despite the small sample sizes and a selection bias towards patients with poor cardiometabolic profile that both limit the generalizability of their findings, the main strength of these studies is the use of polysomnography, the reference standard for the diagnosis and severity assessment of OSA. The frequency of OSA in these two studies from Tunisia is higher than rates between 21% and 74% reported by previous studies in various settings [8]. The wide variance in prevalence rates across studies is mainly due to differences in sensitivity of sleep-study recording techniques and scoring criteria, as well as in the risk profile of study populations. For instance, the cutoff AHI score used for the diagnosis of OSA in various studies ranges between 5 and 15 episodes per hour [8]. Moreover, whereas some studies investigating the prevalence of OSA included patients with AF and various risk profiles, others focused on patients undergoing AF ablation, with mostly longstanding AF resistant to pharmacotherapy and therefore, who are more likely to have OSA [8].

Atrial fibrillation and OSA frequently co-exist due to shared risk factors including obesity, hypertension, and diabetes that are increasingly prevalent in the general population [8,11]. Obstructive sleep apnea is also an important risk factor for the occurrence and progression of AF [8,11,12]. First, acute apnea is associated with an increase in atrial arrhythmogenesis through several mechanisms. Negative intrathoracic pressure swings during obstructed inspiration and causes acute atrial dilation that shortens atrial refractoriness, slows conduction, and increases the occurrence of intermittent conduction delay [8]. Furthermore, the sympathovagal activation caused by obstructed breathing efforts induces acute electrophysiological arrhythmogenic changes that could trigger AF in a vulnerable substrate [8]. Second, in the long-term, OSA is associated with marked atrial structural and electrical changes. Intermittent episodes of deoxygenation and reoxygenation lead to oxidative stress that, along with chronic neurohormonal activation contribute to atrial fibrosis [8]. Repetitive mechanical atrial stretch from recurrent obstructive respiratory episodes also causes myocardial injury and remodeling, as well as regional conduction slowing and reentry [8]. All these OSA-induced changes are exacerbated by concomitant conditions such as obesity, hypertension and diabetes, and ultimately constitute a complex and dynamic arrhythmogenic substrate for AF [8].

The presence and severity of OSA have a substantial impact on AF outcomes. Although the evidence is limited, OSA has been reported as an independent predictor of stroke in patients with AF [13]. Obstructive sleep apnea significantly reduces the effectiveness of rhythm control strategies including antiarrhythmic drug therapy and catheter-based pulmonary vein isolation [14-16]. Additionally, several non-randomized observational studies and meta-analyses showed that treatment of OSA by continuous positive airway pressure (CPAP) may improve success rates after catheter ablation in patients with AF [14,17]. As a result, the 2020 European Society of Cardiology (ESC) guidelines for the diagnosis and management of AF indicate that: 1) opportunistic screening for AF should be considered in patients with OSA (class IIa, level C) and; 2) optimal management of OSA may be considered, to reduce AF incidence, AF progression, AF recurrences, and symptoms (class IIb, level C) [18]. In terms of screening for OSA in patients with AF, whereas the 2016 ESC guidelines for the diagnosis and management of AF recommended that the interrogation for clinical signs of OSA should be considered in all AF patients (class IIa, level C) [19], the 2020 ESC guidelines acknowledge that it remains unclear how and when to test for OSA and implement OSA management in the standard work-up of patients with AF [18].

In the light of this body of knowledge on the association between OSA and AF, and the current related recommendations from international professional societies [18,19], the findings of the studies by Afef Ben Halima and Manel Ben Halima and their colleagues have few implications [9,10]. Considering its potentially high prevalence and its adverse effects, OSA should receive enough attention from physicians managing patients with AF in African settings. There is no specific data on the availability and affordability of polysomnography and CPAP in various African health facilities. Manel Ben Halima *et al*. acknowledged a limited accessibility to polysomnography in their context [9]. The situation is probably similar or even horse in the vast majority of health facilities across the continent which are very resource-constrained. Considering the low affordability and availability of basic medications for cardiovascular disease in Africa [20,21], a more expensive therapy like CPAP would probably not be accessible to a large proportion of patients who need it. In this context, lifestyle interventions for the prevention and control of OSA are of utmost importance.

Indeed, the role of risk factor control targeting hypertension, diabetes, obesity, dyslipidemia, OSA, physical inactivity and alcohol consumption is now recognized as a major component in the management of patients with AF [18]. Patients with AF and OSA should be screened for exacerbating factors such as obesity and alcohol consumption. Alcohol consumption prior to bedtime has been associated with more frequent and longer episodes of hypopnea and apnea in people who snore or have OSA [22]. Furthermore, obesity is strongly associated with OSA and, in more extreme cases, obesity hypoventilation syndrome [11]. There is compiling evidence showing that significant and sustained weight loss, if achieved, is likely to be a useful therapeutic option in the management of OSA [23]. Thus, weight reduction and alcohol cessation should be strongly encouraged in patients with AF and OSA. In a context of limited availability of polysomnography, questionnaires such as the Epworth Sleepiness Scale or the Berlin questionnaire remain very useful in identifying patients with a high probability of OSA and who should consider costless lifestyle interventions. Nevertheless, CPAP should not be overlooked and should rather be offered whenever indicated and available, especially in patients with severe OSA. Such contextualized approaches might contribute in reducing the adverse effects of OSA in patients with AF in resource-limited settings in Africa.
